# Characterization of Fulvic Acid Beverages by Mineral Profile and Antioxidant Capacity

**DOI:** 10.3390/foods8120605

**Published:** 2019-11-22

**Authors:** Monika Swat, Iga Rybicka, Anna Gliszczyńska-Świgło

**Affiliations:** Institute of Quality Science, Poznań University of Economics and Business, al. Niepodległości 10, 61-875 Poznań, Poland; monika.swat@wp.pl (M.S.); anna.gliszczynska-swiglo@ue.poznan.pl (A.G.-Ś.)

**Keywords:** antioxidant capacity, fulvic acids, functional beverage, iron, mineral

## Abstract

The main purpose of the study was to investigate the quality of fulvic acid-based food products. The concentrations of Ca, K, Mg, Na, Cu, Fe, Mn, and Zn, and antioxidant capacities of fulvic acid concentrates and ready-to drink beverages available on the global market were determined. The concentrations of minerals were determined using microwave plasma-atomic emission spectrometry. Antioxidant capacity was expressed as total polyphenol (TP) and flavonoid (TF) contents, the trolox equivalent antioxidant capacity (TEAC) and ferric reducing ability of plasma (FRAP) values. The daily portion of eight out of 14 products realized 45–135% of recommended daily allowance (RDA) for Fe. One of ready-to-drink beverages was also a good source of Mg (about 40% of RDA), and another one of Mn (about 70% of RDA). The concentrations of TP and TF in ready-to-drink beverages varied from 6.5 to 187 µg/mL, whereas in concentrates, from 5886 to 19,844 µg/mL. Dietary supplements or food products with fulvic acids may be a good source of antioxidant polyphenolic compounds and some minerals.

## 1. Introduction

Fulvic acids are natural, water-soluble polymers, which are the ingredients of humic substances defined as “a series of high molecular weight substances, yellow to black in colour, formed as a result of secondary synthesis reactions” [1,2]. They are complex substances without standard chemical formulae, which are present in soil and plants in trace amounts [3,4]. They are formed during the decomposition of decaying plants by microorganisms and they play essential functions in plants; e.g., are responsible for the absorption of nutrients and trace substances. Naturally, fulvic acids contain minerals (more than 70), amino acids, sugars, peptides, nucleic acids, phytochemical compounds, vitamins, and fragments of plant DNA [3]. Most of them occur in ionic form. This means that fulvic acids conduct electricity excellently and improve the absorption of other compounds interacting with them. Moreover, because of ionic minerals, fulvic acids help to increase their bioavailability in plants [5]. Fulvic acids are also chemically reactive because of the presence of many carboxyl and hydroxyl groups [3]. Due to their low molecular weights, they can transport minerals to plant cells in the root, stem, and leaves [6]. They also participate in the carbon cycle, because they are constantly recycled among plants, soil, and water [7].

The results of the studies on fulvic acid properties conducted with plants and plant cells indicate the positive effect of these substances on animal organisms. Kishor et al. [8] suggested that humic substances, consisting of 60–80% fulvic acids, have anticarcinogenic properties. They may be beneficial in cancer therapy due to their heavy metal chelating properties, binding of proteins delivering anticancer drugs, and inhibition of cancer cell proliferation [9,10,11]. Fulvic acids are also good free radical scavengers. They may scavenge superoxide (O_2_^•−^), hypochlorous acid (HOCl), hydrogen peroxide (H_2_O_2_), hydroxyl radicals (^•^OH), peroxynitrite (ONOO^−^), and singlet oxygen (^1^O_2_) [12,13]. Carrasco-Gallardo et al. [14] analysed the results of various clinical studies confirming the effect of fulvic acid consumption on the reduction the symptoms of Alzheimer’s disease. Their studies suggested that fulvic acids can be potentially used to prevent this brain disorder, mainly due to their antioxidant properties. Many studies have provided the evidence that accumulation of oxidative stress products in brain tissue is closely associated with the development of Alzheimer’s disease and antioxidant therapies are one of the promising therapeutic strategies for this disease [15]. It is thought that fulvic acids improve the absorption of iron, making it more bioavailable to bone marrow stem cells for formation of blood [16]. Van Rensburg [17] and Winkler and Gosh [18], in their reviews, listed few studies conducted with both animals and humans over several years on the safety of fulvic acids and their effects on human diseases. In these studies, fulvic acids were applied orally and topically. The studies were carried out using various doses and forms of fulvic acids, and with various durations. The results obtained suggested that fulvic acids are safe for humans; nevertheless, further studies to ensure their safety are still required. The recommended daily dose of fulvic acids for people is not established. Therefore, it is important to determine the optimal dosage of fulvic acids for different age groups to prevent them from overdosing.

Fulvic acids have been primarily used as products supporting the growth of plants and maintaining soil moisture. Currently, the food market has also become interested in them. Considering all the properties of fulvic acids, there is a potential to use them as a new, natural, and valuable food additives or supplements [19]. Due to the structure of fulvic acids and their chelating properties they can help to transport some nutrients, mainly minerals, to cells and remove deeply embedded toxins from the body [16]. Substances constituted up to 20% by fulvic acids have been used in traditional Indian medicine, “Ayurveda,” for medicinal purposes for about 3000 years [20,21]. Fulvic acids intended for human consumption are currently available in concentrated form, ready-to-drink beverages and pills, but their variety on the food market is still small. These products are a potential source of minerals and antioxidant compounds. As far as is known, there are no data characterizing fulvic acid products in the aspects of either mineral profile or antioxidant capacity. Therefore, the objective of this study was to determine the content of selected macroelements and microelements and the antioxidant properties of fulvic acid beverages available on the global market.

## 2. Materials and Methods

### 2.1. Materials

The products were bought in 2015 in on-line stores. The analyses were performed for most of fulvic acid beverages available at that time on the global market. The experiments were repeated for the same products bought in 2018. The total of 14 beverages were included in the study—6 concentrates and 8 ready-to-drink beverages. According to the label description, fulvic acids in concentrated form originated from the Great Salt Lake in Utah (North America) and England. Fulvic acids in ready-to-drink beverages were obtained mainly from an aquatic source and/or soils of North America and South Africa.

### 2.2. Sample Preparation

For determination of minerals, the products tested were diluted with demineralized water (Hydrolab System, Wiślina, Poland). The dilution of ready-to-drink beverages was 10-fold and that of concentrates was 100-fold.

For determination of total phenolics (TP), total flavonoids (TF), and the antioxidant capacities, the products were centrifuged at 14,000× *g* for 5 min (MiniSpin plus centrifuge, Eppendorf, Hamburg, Germany) and diluted with demineralized water if necessary.

### 2.3. Determination of Minerals

The concentrations of Ca, K, Mg, Na, Cu, Fe, Mn, and Zn were determined using microwave plasma-atomic emission spectrometry (Agilent MP-AES 4210) (Agilent Technologies, Melbourne, Australia) according to the method described in detail by Ozbek and Akman [22]. The analytical wavelengths and standard curves for minerals we analysed were: 616.217 nm and 0–50 µg/mL for Ca, 404.414 nm and 0–500 µg/mL for K, 383.829 nm and 0–20 µg/mL for Mg, 330.237 nm and 0–100 µg/mL for Na, 324.754 nm and 0–0.1 µg/mL for Cu, 371.993 nm and 0–10 µg/mL plus 259.940 nm and 10–100 µg/mL for Fe, 403.076 nm and 0–10 µg/mL for Mn, and 213.857 nm and 0–5 µg/mL for Zn. For each determination, at least two calibration curves were prepared, each adjusted to the expected concentration in the sample being analysed. Six determinations were performed for each sample.

### 2.4. Determination of Total Phenolics

The concentration of TP was determined according to Singleton and Rossi [23]. The method was adapted to 48-well microplates. In brief, 0.01 mL of each sample was mixed with 0.05 mL of the Folin–Ciocalteu reagent. After 3 min, 0.15 mL of 20% sodium carbonate and 0.79 mL of demineralized water were added and the solution was mixed. The plate was incubated for 2 h in the dark at room temperature. The absorbance was measured at 765 nm. At least six determinations were performed for each sample. The total content of phenolics was expressed in µg of gallic acid per millilitre of product.

### 2.5. Determination of Total Flavonoids

The concentration of TF was determined according to Karadeniz et al. [24]. The method was adapted to 48-well microplate and (±)-catechin was used as the standard. In brief, 0.10 mL of each sample was mixed with 0.50 mL of demineralized water and 0.03 mL of 5% NaNO_2_. After 5 min, 0.06 mL of 10% AlCl_3_ and 0.2 mL of 1M NaOH were added. After 5 min, 0.11 mL of demineralized water was added and the solution was mixed. The absorbance was measured at 510 nm and corrected for the absorbance of product sample and the absorbance of blank sample. At least six determinations were performed for each sample. The total content of flavonoids was expressed in µg of (±)-catechin per millilitre of the product. 

### 2.6. Determination of the Antioxidant Capacity

The antioxidant capacity of fulvic acid products was determined using the TEAC method with ABTS^•+^ radical cation as described by Re et al. [25]. Moreover, the FRAP assay was carried out by the method of Benzie and Strain [26] with modifications previously described [27].

Briefly, the ABTS^•+^ radical cation was generated by reaction of 0.0077 g of ABTS dissolved in 1.8 mL of demineralized water with 0.2 mL of 0.0066 g/mL potassium persulphate. The reaction mixture was incubated in the dark at room temperature for 16 h [25]. The ABTS^•+^ radical cation working solution was obtained by dilution with methanol to an absorbance about 0.80 at 734 nm. The absorbance was measured 6 min after mixing 0.008 mL of sample with 0.792 mL of the ABTS^•+^ radical cation working solution. The TEAC value was calculated as the ratio of the linear regression coefficient of the calibration curve for five dilutions of the sample and the linear regression coefficient of the trolox standard curve. Three independent determinations were performed for each sample. The activity of each product was expressed as the TEAC value (in μmol of trolox/mL of product).

The FRAP assay is based on the reduction of a ferric 2,4,6-tripyridyl-s-triazine complex (Fe^3+^-TPTZ) to the ferrous form (Fe^2+^-TPTZ) in the presence of antioxidant. A volume of 0.008 mL of sample was added to 0.792 mL of the 10 mM ferric-TPTZ reagent and the increase in absorbance at 593 nm was measured after 8 min. The FRAP value was calculated as the ratio of the linear regression coefficient of the calibration curve for five dilutions of the sample and the linear regression coefficient of the FeSO_4_ × 7 H_2_O standard curve [27]. Three independent determinations were performed for each sample. Activity of each product was expressed as the FRAP value (μmol of Fe^2+^/mL).

### 2.7. Statistical Analysis

Statistical analyses were carried out using Statistica 12.0 software (2013; StatSoft, Inc., Tulsa, OK, USA). All data were submitted to one-way analysis of variance (ANOVA). The significances of differences between mean values obtained for products were determined by the least significant differences tests at α = 0.05. The comparison of data in Table 1, Table 2 and Table 3 is category-separated for clarity of data presentation.

## 3. Results and Discussion

Each producer of a fulvic acid beverage declared that they used a standard, repeatable, and controlled process to manufacture each batch of their product(s). On the other hand, they usually stated that some differences in the concentration of fulvic acids and individual trace substances may be observed because the starting material is a completely natural material, not subjected to any laboratory treatment. Because of this, products from different years (2015 and 2018) were regarded as independent samples. As products selected were from different categories (concentrates and ready-to-drink beverages); all results were expressed both per millilitre of the product and in regard to daily intake calculated for the maximum portion suggested by producers.

Table 1 and Table 2 present the concentrations of macroelements and microelements in fulvic acid beverages. The percentages of the realization of recommended daily allowances (RDAs for Ca, Mg, Cu, Fe, Mn, and Zn) and adequate intakes (AIs for K and Na) were calculated for the maximum daily portions suggested by the producers. The maximum portions were: 1.45 mL, 0.7 mL, and 2.8 mL for concentrates 1, 2, and 3, respectively. For ready-to-drink beverages 4, 5, 6, and 7 they were 28 mL, 28 mL, 16 mL, and 500 mL, respectively. The percentages of the realizations of RDA/AI were calculated according to American and Polish recommendations for middle-aged (40 years old) healthy woman [28,29] and are also presented in Table 1 and Table 2. The RDA or AI values were: 1000 mg for Ca, 4700 mg for K, 320 mg for Mg, 1500 mg for Na, 0.9 mg for Cu, 18 mg for Fe, 1.8 mg for Mn, and 8 mg for Zn. The choice of another model person (man or child) would change %RDA or %AI values, but would not change the mutual relationships between the products being tested.

It was found that fulvic acid beverages differed within categories of products in the concentrations of minerals tested (*p* < 0.05), and eight out 14 of them were an excellent source of Fe. Although further studies on the bioavailability of Fe from these products are necessary, the content of this mineral needs to be underlined. The content of Fe in two out of three kinds of concentrates (product 1 and 2) was between 12 and 16 mg in 1 mL of the product, whereas for two kinds of ready-to-drink products was between 0.8 and 1.2 mg in 1 mL. One kind of concentrate and two ready-to-drink beverages contained trace amounts of this element. The maximum daily portion of products 1, 4, and 6 realized more than 100% of the RDA for Fe. Product 2 was also a good source of this element and realized approximately 45–50% of dietary requirement for iron. The presence of a significant amount of iron in commercially available fulvic acids was also confirmed by other scientists who studied the effect of fulvic acids on the growth of tomatoes, cucumbers, sunflowers, and lemon trees [30,31,32,33]. The important sources of other minerals were: product 3—Mg (approximately 40% of RDA); product 6—Mn and K (approximately 70% or 40% of RDA or AI, respectively); and product 7—Na (approximately 18% of AI).

Grant et al. [34] also underlined the potential benefit of fulvic acid dietary supplements as a source of some minerals. They investigated the elemental composition of four fulvic acid dietary supplements in a liquid (two fulvic acid mineral waters and fulvic mineral complex) and capsule form (fulvic acid dietary supplement). The origins of fulvic acids in the samples which they tested were not presented. Their results also confirmed the presence of Mg—the range of 0.89–7.20 mg/g, which was not as wide as in our study (from below 0.01 to 50 mg/mL). The content of Fe was also significantly higher for most of the products in our study (<0.01–16 mg/mL) than for samples from Grant et al.’s study (0.15–0.98 mg/g) [34]. On the other hand, our products mostly contained smaller amounts of Zn (Grant et al.: 0.16–2.2 mg/g; our study: <0.01–0.06 mg/mL), Mn (Grant et al.: 0–1.55 mg/g; our study: <0.01–0.21 mg/mL), and Cu (Grant et al.: 0.022–0.034 mg/g; our study: <0.01 mg/mL). The differences between products from different years or the same category or between our study and study of Grant et al. [34] may result from variety of fulvic acid materials, their origin from soils and plants growing in various conditions, and extraction methods used by producers to obtain them. Moreover, the compositions of the final products, including the amounts of fulvic acids, differ between producers.

Although in vitro assays for determination of the antioxidant activity of compounds do not reflect in vivo conditions, their application may give some view of the potential antioxidant activities in vivo. Therefore, Table 3 presents the antioxidant capacities of the products we tested, expressed as the TP, TF, FRAP, and TEAC values. It was found that fulvic acid beverages differed within categories of products in polyphenol and flavonoid contents, and in the TEAC and FRAP values (*p* < 0.05). TP content ranged from 12–67 μg/mL in ready-to-drink beverages to 12,814–19,844 μg/mL in concentrates. The concentration of TF in drinks ranged from 6.5 to 187 μg/mL, and in concentrates from 5886 to 7321 μg/mL. Only two kinds of concentrates and one kind of ready-to-drink had antioxidant activity expressed as the FRAP and/or TEAC values. Product 3 had no antioxidant activity. As it can be expected, concentrates had up to 1650-fold more antioxidants than ready-to-drink beverages. However, taking into account the maximum daily portion suggested by the producer, the differences were much lower (up to 93-fold for TP, 58-fold for TF, and 6.2-fold for FRAP value). The daily portion of product 7 was an even better source of TF than the daily portion of concentrates. Moreover, the concentrations of TP or TF in fulvic acid concentrates, and their TEAC and FRAP values, were much higher than reported for many polyphenol-rich beverages, such as red wine, fruit juices, antioxidant-enriched juices, and ice-teas [35,36,37,38].

Fulvic acids in a liquid form can be a good natural substitute for artificial isotonic beverages widely available on the global food market or an interesting option for people with iron deficiency. Nowadays, the market of beverages strongly benefits from the healthy lifestyles of contemporary consumers. Consumers tend to eat more food from the categories “natural,” “organic,” or “functional.” Lal [39] emphasized that the sector of functional beverages could be the category where consumers look for innovations. Thus, fulvic acids drinks, due to their antioxidant properties and high contents of important microelements and macroelements (e.g., Fe and Mg), could be perceived as the functional beverages that do not contain artificial additives, such as sweeteners, colorants, preservatives, and flavour enhancers. They could be an interesting opportunity for both producers and consumers and may find a high acceptance among health-conscious people. 

## 4. Conclusions

Nowadays, there is a growing interest among consumers for products made from natural ingredients, not containing preservatives, and having beneficial effects on humans. Besides economic factors, the taste and the health awareness of consumers on the importance of nutrients supplied with food to the human body have a great impact on the food market, including beverages. 

The results of the present study on beverages containing fulvic acids, available in the form of concentrates and ready-to-drink beverages, showed that they may contain substantial amounts of Fe (up to about 130% of RDA), Mg (up to about 40% of RDA), and Mn (up to about 70% of RDA). They can also be a good source of polyphenolic compounds (up to about 19.8 mg/mL) with high antioxidant activity. Therefore, they may become interesting and valuable food products or food ingredients with potential effects on human health. 

## Figures and Tables

**Table 1:** The content of macroelements in fulvic acid beverages.

Category of Product		Ca		K		Mg		Na	
		I ^1^	II ^1^	I	II	I	II	I	II
**CONCENTRATE**									
**1**	mg/mL mg/daily portion ^2^ %RDA/AI ^3^	0.99 ± 0.01 ^c^	2.09 ± 0.11 ^b^	24.0 ± 0.2 ^a^	24.1 ± 0.6 ^a^	1.09 ± 0.02 ^c^	1.05 ± 0.02 ^c^	3.76 ± 0.14 ^a^	3.62 ± 0.15 ^a,b^
		1.4	3.0	34	35	1.6	1.5	5.5	5.3
		0.1%	0.3%	0.7%	0.7%	0.5%	0.5%	0.4%	0.4%
**2**		0.92 ± 0.05 ^c,d^	0.91 ± 0.03 ^d^	23.0 ± 0.4 ^b^	23.0 ± 0.4 ^b^	1.46 ± 0.02 ^b^	1.46 ± 0.03 ^b^	3.50 ± 0.21 ^a,b^	3.32 ± 0.16 ^b^
		0.6	0.6	16	16	1.0	1.0	2.5	2.3
		<0.1%	<0.1%	0.3%	0.3%	0.3%	0.3%	0.2%	0.2%
**3**		2.57 ± 0.17 ^a^	2.62 ± 0.09 ^a^	1.82 ± 0.17 ^c^	1.98 ± 0.07 ^c^	50 ± 2.3 ^a^	48 ± 2.2 ^a^	2.12 ± 0.06 ^c^	2.05 ± 0.05 ^c^
		7.2	7.3	5.1	5.5	139	135	5.9	5.7
		0.7%	0.7%	0.1%	0.1%	43%	42%	0.4%	0.4%
**READY-TO-DRINK**									
**4**	mg/mL mg/daily portion %RDA/AI	0.20 ± 0.01 ^c^	0.33 ± 0.01 ^b^	0.28 ± 0.01 ^b^	0.18 ± 0.00 ^c^	0.06 ± 0.01 ^b^	0.07 ± 0.01 ^a^	0.58 ± 0.02 ^a^	0.57 ± 0.05 ^a,b^
		5.6	9.2	7.8	5.0	1.7	2.0	16	16
		0.6%	0.9%	0.2%	0.1%	0.5%	0.6%	1.1%	1.1%
**5**		0.12 ± 0.01 ^d^	0.20 ± 0.00 ^c^	<0.01	<0.01	<0.01	<0.01	0.53 ± 0.04 ^a,b^	0.52 ± 0.03 ^b^
		3.4	5.6	0	0	0.3	0.3	15	15
		0.3%	0.6%	0	0	0.1%	0.1%	1%	1%
**6**		0.38 ± 0.03 ^a^	0.37 ± 0.01 ^a^	2.46 ± 0.06 ^a^	2.56 ± 0.07 ^a^	0.10 ± 0.00 ^a^	0.10 ± 0.00 ^a^	0.58 ± 0.04 ^a^	0.62 ± 0.06 ^a^
		6.1	5.9	40	41	1.6	1.6	9.3	9.9
		0.6%	0.6%	0.8%	0.9%	0.5%	0.5%	0.6%	0.7%
**7**		0.02 ± 0.00 ^e^	0.02 ± 0.00 ^e^	0.04 ± 0.00 ^d^	0.04 ± 0.00 ^d^	<0.01	<0.01	0.53 ± 0.01 ^b^	0.54 ± 0.01 ^b^
		10	10	20	20	0	0	265	270
		1%	1%	0.4%	0.4%	0	0	18%	18%

^1^—Production years: 2015 and 2018; ^2^—the maximum daily intake, ^3^—the percentage of recommended daily allowance (RDA)/adequate intake (AI) realized by maximum daily portion; a–e: significant differences (*p* < 0.05) between mean values for each mineral within the product categories are indicated by different letters.

**Table 2:** The content of microelements in fulvic acid beverages.

Category of Product		Cu		Fe		Mn		Zn	
		I ^1^	II ^1^	I	II	I	II	I	II
**CONCENTRATE**									
**1**	mg/mL mg/daily portion ^2^ %RDA ^3^	<0.01	<0.01	16.0 ± 1.5 ^a^	14.0 ± 1.0 ^a,b^	0.16 ± 0.00 ^b^	0.16 ± 0.00 ^b^	0.06 ± 0.00 ^a^	0.05 ± 0.00 ^a,b^
		0	0	23	20	0.23	0.23	0.09	0.07
		0	0	129%	113%	13%	13%	1.1%	1.0%
**2**		<0.01	<0.01	13.0 ± 1.1 ^b^	12.0 ± 1.1 ^b^	0.20 ± 0.01 ^a^	0.21 ± 0.00 ^a^	0.04 ± 0.00 ^b^	0.04 ± 0.00 ^b^
		0	0	9.0	8.1	0.14	0.15	0.03	0.03
		0	0	50%	45%	7.8%	8.2%	0.4%	0.4%
**3**		<0.01	<0.01	0.04 ± 0.00 ^c^	0.04 ± 0.00 ^c^	<0.01	<0.01	0.02 ± 0.00 ^c^	0.02 ± 0.00 ^c^
		0	0	0.11	0.11	0	0	0.06	0.06
		0	0	0.6%	0.6%	0	0	0.7%	0.7%
**READY-TO-DRINK**									
**4**	mg/mL mg/daily portion %RDA	<0.01	<0.01	0.83 ± 0.04 ^b^	0.87 ± 0.04 ^b^	<0.01	<0.01	0.01 ± 0.00 ^b^	0.01 ± 0.00 ^b^
		0	0	23	24	0	0	0.28	0.28
		0	0	129%	135%	0	0	3.5%	3.5%
**5**		<0.01	<0.01	<0.01	<0.01	<0.01	<0.01	0.02 ± 0.00 ^a^	0.01 ± 0.00 ^b^
		0	0	0	0	0	0	0.56	0.28
		0	0	0	0	0	0	7%	3.5%
**6**		<0.01	<0.01	1.19 ± 0.01 ^a^	1.20 ± 0.01 ^a^	0.08± 0.00	0.08 ± 0.00	0.01 ± 0.00 ^b^	0.01 ± 0.00 ^b^
		0	0	19	19	1.28	1.28	0.16	0.16
		0	0	106%	107%	71%	71%	2%	2%
**7**		<0.01	<0.01	<0.01	<0.01	<0.01	<0.01	<0.01	<0.01
		0	0	0	0	0	0	0	0
		0	0	0	0	0	0	0	0

^1^—Production years: 2015 and 2018; ^2^—the maximum daily intake, ^3^—the percentage of RDA realized by maximum daily portion; a–c: significant differences (*p* < 0.05) between mean values for each mineral within product categories are indicated by different letters.

**Table 3:** The antioxidant capacities expressed as the total polyphenol (TP) and total flavonoid (TF) (µg/mL), ferric reducing ability of plasma (FRAP) and trolox equivalent antioxidant capacity (TEAC) (µmol/mL) of fulvic acid beverages.

Category of Product		TP		TF		FRAP		TEAC	
		I ^1^	II ^1^	I	II	I	II	I	II
**CONCENTRATE**									
**1**	µg/mL µg/daily portion ^2^	15,762 ± 140 ^c^	16,457 ± 442 ^b^	7321 ± 152 ^a^	5886 ± 228 ^b^	309 ± 15 ^a^	235 ± 6 ^b^	141 ± 5 ^b^	117 ± 13 ^c^
		22,855	23,863	10,615	8535	448	341	204	170
**2**		12,814 ± 175 ^d^	19,844 ± 231 ^a^	6167 ± 163 ^b^	7113 ± 270 ^a^	309 ± 16 ^a^	338 ± 18 ^a^	150 ± 10 ^b^	172 ± 9 ^a^
		8970	13,891	4317	4979	216	237	105	120
**3**		n.d.	n.d.	n.d.	n.d.	n.d.	n.d.	n.d.	n.d.
		n.d.	n.d.	n.d.	n.d.	n.d.	n.d.	n.d.	n.d.
**READY-TO-DRINK**									
**4**	µg/mL µg/daily portion	44.7 ± 1.3 ^b^	67.3 ± 2.8 ^a^	17.1 ± 0.4 ^e^	24.4 ± 0.6 ^d^	n.d.	n.d.	n.d.	n.d.
		1252	1884	479	683	n.d.	n.d.	n.d.	n.d.
**5**		12.0 ± 2.6 ^e^	12.0 ± 0.5 ^e^	6.5 ± 0.5 ^g^	8.1 ± 0.3 ^f^	n.d.	n.d.	n.d.	n.d.
		336	336	182	227	n.d.	n.d.	n.d.	n.d.
**6**		22.0 ± 1.7 ^c^	16.0 ± 0.5 ^d^	187 ± 10 ^a^	135 ± 20 ^b^	4.8 ± 0.7	4.5 ± 0.1	n.d.	n.d.
		352	256	2992	2160	77	72	n.d.	n.d.
**7**		22.2 ± 0.8 ^c^	21.4 ± 1.0 ^c^	28.4 ± 0.6 ^c^	29.3 ± 0.8 ^c^	n.d.	n.d.	n.d.	n.d.
		11,100	10,700	14,200	14,650	n.d.	n.d.	n.d.	n.d.

^1^—production years: 2015 and 2018; ^2^—the maximum daily intake, n.d.—not detected; a–g: significant differences (*p* < 0.05) between mean values for TP/TF/FRAP/TEAC within a product category were indicated by different letters.
